# A Novel Closed-Loop Single-Channel Time Division Multiplexing Detection Circuit for Hemispherical Resonator Gyroscope

**DOI:** 10.3390/mi16030273

**Published:** 2025-02-27

**Authors:** Qi Wang, Weinan Xie, Boqi Xi, Hanshi Li, Guoxing Yi

**Affiliations:** Space Control and Inertial Technology Research Center, Harbin Institute of Technology, Harbin 150001, China; wangqi95@stu.hit.edu.cn (Q.W.); xibq@hit.edu.cn (B.X.); lihanshi99@stu.hit.edu.cn (H.L.); ygx@hit.edu.cn (G.Y.)

**Keywords:** hemispherical resonator gyroscope, time division multiplexing, gain stability, phase delay

## Abstract

The vector control method is applied to a whole angle hemispherical resonator gyroscope (HRG). The detection and control of the resonator vibration state are implemented using orthogonal *X*/*Y* channels. However, the performance of the HRG is limited by the asymmetry in the gain and phase delay of *X*/*Y* channels. To address these issues, a novel detection circuit is proposed. The circuit leverages the closed-loop characteristics to achieve symmetry and stability in the *X*/*Y* channel gain while simultaneously eliminating phase delays within the loop. Firstly, a closed-loop single-channel time division multiplexing circuit is designed to overcome the deficiencies of the traditional dual-channel circuit. Secondly, a model is developed to analyze the time division detection errors, and an improved demodulation method is proposed to mitigate detection errors. Lastly, experimental results demonstrate that the designed circuit successfully suppresses drift in both gain and phase delay within the loop, confirming the effectiveness of the proposed solution in enhancing the performance of the HRG.

## 1. Introduction

The hemispherical resonator gyroscope has become a prominent focus in current inertial sensor research due to its simple structure, high reliability, and the capability to achieve both high precision and high dynamic measurement performance [1,2]. The superior attributes provide extensive application prospects in areas such as aerospace navigation, robotics, and autonomous systems [3,4,5,6]. A HRG mainly consists of four parts: the hemispherical resonator, flat electrodes, metal housing, and a circuit. The hemispherical resonator is made from fused silica material and is fabricated into a rough shape with a thickness of approximately 1 mm and a diameter of around 20 mm through ultra-precision machining. Subsequent physical and chemical polishing eliminates the machining defects [7,8]. Ion beam leveling is then applied to ensure that the resonator circumferential resonant frequencies are closely matched [9]. Finally, a metal coating is applied to the resonator to provide electrical conductivity. The flat electrodes are first coated with a metal layer to form effective electrodes with the resonator rim. Laser etching is then used to uniformly etch the flat electrodes into equal-sized sectors, with each sector electrically insulated from the others. The metal housing is typically made from Kovar alloy material. The resonator, planar electrodes, and metal housing are then welded together to form the core sensitive element. A matching circuit is designed and implemented based on the functional requirements to complete the fabrication of the HRG. To attain the optimal performance of the HRG, precise calibration and compensation of various errors are typically required. These errors primarily originate from two sources: errors introduced during the manufacturing and assembly processes of the HRG and errors within the signal detection and drive circuit [10,11,12]. Circuit-related errors are mainly manifested as asymmetries in the *X*/*Y* channels and phase delays between the vibration signals and control signals. Asymmetries in the *X*/*Y* channels may result from differences in amplifier gain or filtering characteristics, leading to unbalanced signal amplitudes and phase shifts, thereby causing measurement biases. Additionally, phase delays between the vibration and control signals may arise from signal processing delays and propagation delays in electronic components. These phase delays can disrupt the intended feedback mechanisms, thereby affecting the ability to accurately track the rotation of the carrier.

In [13], the authors employ a self-precession method to observe the relationship between standing waves at the *X*/*Y* axis positions and the control forces, thereby determining gain errors between different channels. This online identification approach demonstrates environmental adaptability. However, it fails to account for phase errors and overlooks calculation inaccuracies in vibration states when gain asymmetry is present, thereby compromising the method precision. In [14], a technique is introduced for calibrating detection gain errors within a closed-loop system through high-speed constant input testing. While effective in this context, the method fails to consider that the detection serves as a feedback loop in the control system. Consequently, any feedback errors induce drive errors, resulting in angle output discrepancies that encompass control errors. This limitation hinders the accurate identification of error parameters. In [15], the authors model systems with mismatched detection and drive gains. They propose an identification method based on modal reversal; however, this approach requires accurate quality factors for the *X* and *Y* axes as references. Since quality factors are temperature-sensitive and cannot be measured in real time during HRG operation, the method is inherently limited. In [16], it provides a detailed derivation of an asymmetry model within the detection pathway, achieving high accuracy. Nonetheless, the method is restricted to offline identification, limiting its practicality for real-time applications. In References [17,18], a relationship is established between error terms and angle velocity output errors, employing an iterative approach to eliminate error terms from the output and subsequently determine error parameters. However, as output errors diminish to the level of noise, the convergence of error parameters becomes ineffective, posing challenges for maintaining accuracy. In References [19,20], the impact of phase delay on the scaling factor of force to rebalance the HRG is analyzed. The authors mitigate the scaling factor nonlinearity by compensating for phase delay. This method is exclusively applicable to force to rebalance mode and lacks generalizability to the whole angle mode, thereby limiting its broader utility. In [21], the authors conduct temperature gradient experiments to identify phase errors at various temperatures. They employ fitting functions to develop a model that correlates temperature with phase error. However, this approach necessitates extended temperature testing periods, which are impractical for large-scale manufacturing. Additionally, the ambiguous nature of the temperature model undermines the ability to ensure consistent performance across the entire temperature range during actual operation. In References [22,23], a time division multiplexing detection and drive method is introduced, effectively addressing the consistency issues related to gain and phase delay. However, the method lacks effective solutions for gain or phase delay drift caused by temperature fluctuations, resulting in a significant gap in maintaining system reliability under varying thermal conditions.

Previous studies have primarily encountered two significant issues. A primary concern is that addressing the asymmetry between the *X*/*Y* channels has largely been limited to either the drive circuit or the detection circuit. When multiple asymmetrical errors are present within the system, the proposed methods are unable to effectively decouple these various errors. Additionally, existing approaches lack efficient mechanisms to suppress parameter drift. The root cause of these challenges lies in the traditional detection and drive circuits, which operate the *X*/*Y* channels independently and in an open-loop configuration. To overcome these limitations, this paper introduces a closed-loop single-channel time division multiplexed circuit. By employing analog switches, the *X*/*Y* channel signals are routed through the same circuit at different time intervals, ensuring consistency between the channels. Furthermore, the vibration signals to be detected are reconstructed within the closed-loop framework, leveraging the inherent ability of closed-loop systems to resist parameter drift and meet stability requirements. The design effectively stabilizes the gain and reduces phase delay in both the *X*/*Y* channels.

The remainder of this paper is organized as follows. Firstly, the vibration state observation and control methods for the HRG are introduced. Secondly, a closed-loop single-channel time division multiplexed circuit structure is designed, and the mathematical model for the signals to be demodulated is derived. Furthermore, detection errors caused by asynchronous sampling are subsequently analyzed, and an optimized demodulation method based on the underlying error mechanisms is proposed. Finally, experimental verification validates the effectiveness of the designed circuit and methods.

## 2. HRG Detection and Drive Principle

### 2.1. HRG Dynamics Model

**Figure 1:**
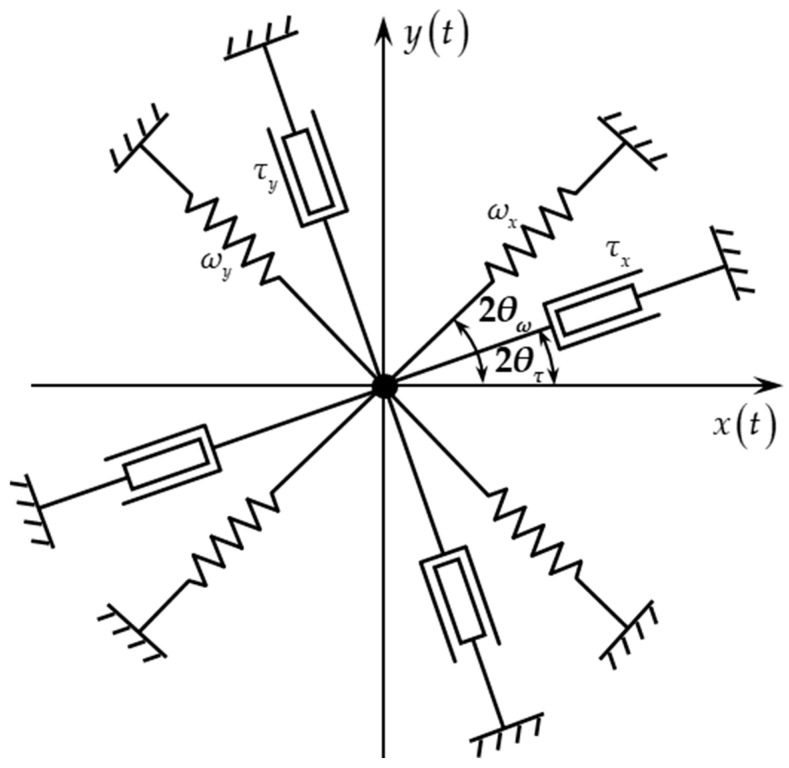
Equivalent model of hemispherical resonator.

As shown in the Figure 1, in the absence of external angular velocity input, the ideal hemispherical resonator model can be equated to a 2-DoF spring-damped model.

For analytical convenience, the resonator intrinsic acceleration, higher-order terms of the external angular velocity and the derivative terms of the external angular velocity input are neglected; the dynamical equations can be expressed as follows:

(1) $$\left\{\begin{array}{l} \ddot{x}+\frac{1}{\tau }\dot{x}+\omega_{0}^{2}x=-2c\Omega_{z}\dot{y}+F_{x}\\ \ddot{y}+\frac{1}{\tau }\dot{y}+\omega_{0}^{2}y=2c\Omega_{z}\dot{x}+F_{y} \end{array}\right.$$

where $F_{x}$, $F_{y}$ are the excitation force in the *X*/*Y* axes, τ is the vibration mechanical time constant, and $\omega_{0}$ is the resonant angular frequency.

In the ideal case, damping and stiffness are fully decoupled between the different axes. Due to the anisotropy of the material parameters of the resonator during the manufacturing process, the actual stiffness and damping have coupling between the *X*/*Y* axes, and the vibration dynamics equations can be expressed as follows [24]:

(2) $$\left\{\begin{array}{l} \ddot{x}+\left(\frac{1}{\tau_{x}}+\frac{1}{\tau_{y}}\right)\dot{x}+\left(\frac{1}{\tau_{x}}-\frac{1}{\tau_{y}}\right)\left(\dot{x}\cos4\theta_{\tau }+\dot{y}\sin4\theta_{\tau }\right)+\frac{\omega_{x}^{2}+\omega_{y}^{2}}{2}x+\frac{\omega_{x}^{2}-\omega_{y}^{2}}{2}\left(x\cos4\theta_{\omega }+y\sin4\theta_{\omega }\right)=-2c\Omega \dot{y}+F_{x}\\ \ddot{y}+\left(\frac{1}{\tau_{x}}+\frac{1}{\tau_{y}}\right)\dot{y}-\left(\frac{1}{\tau_{x}}-\frac{1}{\tau_{y}}\right)\left(-\dot{x}\sin4\theta_{\tau }+\dot{y}\cos4\theta_{\tau }\right)+\frac{\omega_{x}^{2}+\omega_{y}^{2}}{2}y-\frac{\omega_{x}^{2}-\omega_{y}^{2}}{2}\left(-x\sin4\theta_{\omega }+y\cos4\theta_{\omega }\right)=2c\Omega \dot{x}+F_{y} \end{array}\right.$$

where $\tau_{x}$, $\tau_{y}$ are the vibration mechanical time constant in the damping axes, $\theta_{\tau }$ is the damping axis azimuth, $\omega_{x}$, $\omega_{y}$ are the resonant angular frequency in the stiffness axes, and $\theta_{\omega }$ is the stiffness axis azimuth.

### 2.2. Vibration Signal Demodulation

When the resonator begins to vibrate in the form of four antinodes, the trajectory of the mass block in the spring-damped model forms an ellipse. The parametric equation of elliptical motion can be expressed as follows [25]:

(3) $$\left\{\begin{array}{l} D_{x}=a\cos2\theta \cos\left(\omega_{\theta }t+\psi_{0}\right)-q\sin2\theta \sin\left(\omega_{\theta }t+\psi_{0}\right)\\ D_{y}=a\sin2\theta \cos\left(\omega_{\theta }t+\psi_{0}\right)+q\cos2\theta \sin\left(\omega_{\theta }t+\psi_{0}\right) \end{array}\right.$$

where *a* is the primary standing wave amplitude, *q* is secondary standing wave amplitude, θ is the standing wave azimuth, $\omega_{\theta }$ is the resonant angular frequency of the resonator at the current standing wave azimuth, and $\psi_{0}$ is the initial phase of vibration.

The parameter in Equation (3) receives the anisotropic determination of Equation (2) and undergoes a slow change with time. The signal containing $\omega_{\theta }$ is treated as the carrier and *a*, *q*, θ as the fundamental. I/Q demodulation is used to demodulate the resonator vibration information. The reference signal for I/Q demodulation can be expressed as follows:

(4) $$\left\{\begin{array}{l} R_{c}=2\cos(\omega_{\theta }t+\psi )\\ R_{s}=2\sin(\omega_{\theta }t+\psi ) \end{array}\right.$$

where ψ is the initial phase of demodulation signals.

The primary demodulation results can be expressed as follows:

(5) $$\left\{\begin{array}{l} C_{x}=LPF\left\{R_{c}D_{x}\right\}=a\cos2\theta \cos\delta +q\sin2\theta \sin\delta \\ S_{x}=LPF\left\{R_{s}D_{x}\right\}=a\cos2\theta \;\sin\delta -q\sin2\theta \cos\delta \\ C_{y}=LPF\left\{R_{c}D_{y}\right\}=a\sin2\theta \cos\delta -q\cos2\theta \sin\delta \\ S_{y}=LPF\left\{R_{s}D_{y}\right\}=a\sin2\theta \sin\delta +q\cos2\theta \cos\delta \end{array}\right.$$

where *LPF*{·} is the low-pass filter, $\delta =\psi -\psi_{0}$.

For a more intuitive representation of the current resonator vibration state, the secondary demodulation results can be expressed as follows:

(6) $$\left\{\begin{array}{l} E={C_{x}}^{2}+{S_{x}}^{2}+{C_{y}}^{2}+{S_{y}}^{2}=a^{2}+q^{2}\\ Q=2(C_{x}S_{y}-C_{y}S_{x})=2aq\\ S=2(C_{x}C_{y}+S_{x}S_{y})=\left(a^{2}-q^{2}\right)\sin4\theta \\ R={C_{x}}^{2}+{S_{x}}^{2}-{C_{y}}^{2}-{S_{y}}^{2}=\left(a^{2}-q^{2}\right)\cos4\theta \\ L=2(C_{x}S_{x}+C_{y}S_{y})=\left(a^{2}-q^{2}\right)\sin2\delta \end{array}\right.$$

where *E* is the energy of the resonator and *Q* characterizes the quadrature wave vibration amplitude. The normative operation of the HRG necessitates continuous energy replenishment to maintain the primary wave amplitude *a*, while suppressing *Q* to zero minimizes the quadrature wave amplitude *q*. *S* and *R* determine the standing wave azimuth θ for HRG output and provide a reference for control force vector assignment. In phase-locked loops, *L* quantifies the phase difference between reference signals and the primary wave vibration, thereby facilitating the demodulation and modulation signals.

### 2.3. Vibration State Control of HRG

The drive is spatially categorized as being in the same direction or orthogonal to the primary standing wave and temporally categorized as being in the same direction or orthogonal to the vibration. The different combinations of time and space that form the four vector drive forces can be expressed as follows:

(7) FaFqFθFδ=Refae−iωθt+ψnaReifqe−iωθt+ψnqRefθe−iωθt+ψnqReifδe−iωθt+ψna

where $f_{a}$, $f_{q}$, $f_{\theta }$, and $f_{\delta }$, are the scalar magnitudes of the corresponding vector drive forces, $n_{a}$ and $n_{q}$ are the directional vectors of the primary wave and the secondary wave, Re[·] is the real part, and *i* is imaginary unit.

The fully decoupled four drive forces can control the vibration state of the resonator, and the dynamic equations can be expressed as follows [26]:

(8) $$\left\{\begin{array}{ll} \dot{E} & \approx -\left[\left(\frac{1}{\tau_{x}}+\frac{1}{\tau_{y}}\right)+\left(\frac{1}{\tau_{x}}-\frac{1}{\tau_{y}}\right)\cos4\left(\theta -\theta_{\tau }\right)\right]E-\frac{\sqrt{E}}{\omega_{\theta }}f_{a}\\ \dot{Q} & \approx -\left(\frac{1}{\tau_{x}}+\frac{1}{\tau_{y}}\right)Q-\left(\omega_{x}-\omega_{y}\right)\sin4\left(\theta -\theta_{\omega }\right)E+\frac{\sqrt{E}}{\omega_{\theta }}f_{q}\\ 2\dot{\theta } & \approx -2c\Omega +\frac{1}{2}\left(\frac{1}{\tau_{x}}-\frac{1}{\tau_{y}}\right)\sin4\left(\theta -\theta_{\tau }\right)+\frac{1}{2}\left(\omega_{x}-\omega_{y}\right)\cos4\left(\theta -\theta_{\omega }\right)\frac{Q}{E}-\frac{f_{\theta }}{2\omega_{\theta }\sqrt{E}}\\ \dot{\delta } & \approx \dot{\psi }+\frac{1}{2}\left(\omega_{x}-\omega_{y}\right)\cos4\left(\theta -\theta_{\omega }\right)+\frac{1}{2}\left(\frac{1}{\tau_{x}}-\frac{1}{\tau_{y}}\right)\sin4\left(\theta -\theta_{\tau }\right)\frac{Q}{E}+\frac{f_{\delta }}{2\omega_{\theta }\sqrt{E}} \end{array}\right.$$

When the *Q* has been suppressed close to zero by the quadrature loop, the velocity of the standing wave azimuth without any angular velocity input can be expressed as follows:

(9) $$2\dot{\theta }\approx \frac{1}{2}\left(\frac{1}{\tau_{x}}-\frac{1}{\tau_{y}}\right)\sin4\left(\theta -\theta_{\tau }\right)-\frac{f_{\theta }}{2\omega_{\theta }a}$$

The circumferential drift due to anisotropy of damping becomes the main error. Rotating modulation of standing waves is the dominant compensation method for suppressing damping drift. The method applies a constant velocity drive force to sustain the precession of the standing wave [26]. The precession transforms the azimuth-dependent circumferential drift into a time-dependent periodic drift. The actual output requires removal of the precession velocity, and the drift is averaged to zero under the integral corresponding to the period. The method is realized on the basis of applying an accurate drive force, which would otherwise additionally cause HRG output error.

## 3. HRG Closed-Loop Time Division Multiplexing Detection and Drive Circuit Design

### 3.1. Conventional Dual-Channel Detection and Drive Circuit

The mechanical signal of the resonator vibration is reflected as a change in capacitance between the resonator lip and the flat plate electrode. The vibration signal is converted to an alternating current signal by the DC high voltage applied to the resonator. The function of the detection circuit is to amplify the weak current signal for ADC conversion and demodulation [27]. Conventional capacitive-voltage conversion circuits typically incorporate charge amplifiers or transimpedance amplifiers as components [28]. The circuit operates in open-loop mode, allowing circuit gain and phase delay to be calibrated only at a fixed operating point. Consequently, variations in ambient temperature or changes in the vibration signal frequency can cause gain and delay drifts, leading to deviations in the acquired signal. Additionally, discrepancies in the device parameters of the *X*/*Y* channels result in asymmetry between the channels.

The digital controller generates the digital control force of the corresponding loop based on the demodulation result. The driver circuit amplifies the control force using an amplifier and applies it to the flat electrodes to control the vibration state. However, the driver circuit is also subject to previously mentioned issues, including drifting circuit parameters and asymmetry of the *X*/*Y* drive channels.

The above asymmetry leads to detection or drive errors, causing the drive force applied to the resonator to deviate from its intended value. Specifically, the drive forces from the amplitude and quadrature loops leak into the velocity loop, resulting in additional standing wave precession. Furthermore, when employing the rotating modulation compensation method proposed in the previous section, the drive force of the velocity loop itself fails to ensure circumferential consistency. The velocity fluctuations in the circumferential direction cause the HRG to produce periodic output errors. Both impair HRG bias instability and scale factor nonlinearity.

Time division multiplexing involves transmitting the *X*/*Y* channel drive or detection signals through the same circuits separately at different time intervals. This approach effectively mitigates the asymmetry between different channels. However, open-loop parameter drift within the circuit persists due to environmental changes.

### 3.2. Novel Closed-Loop Single-Channel Time Division Multiplexing Circuit Design

**Figure 2:**
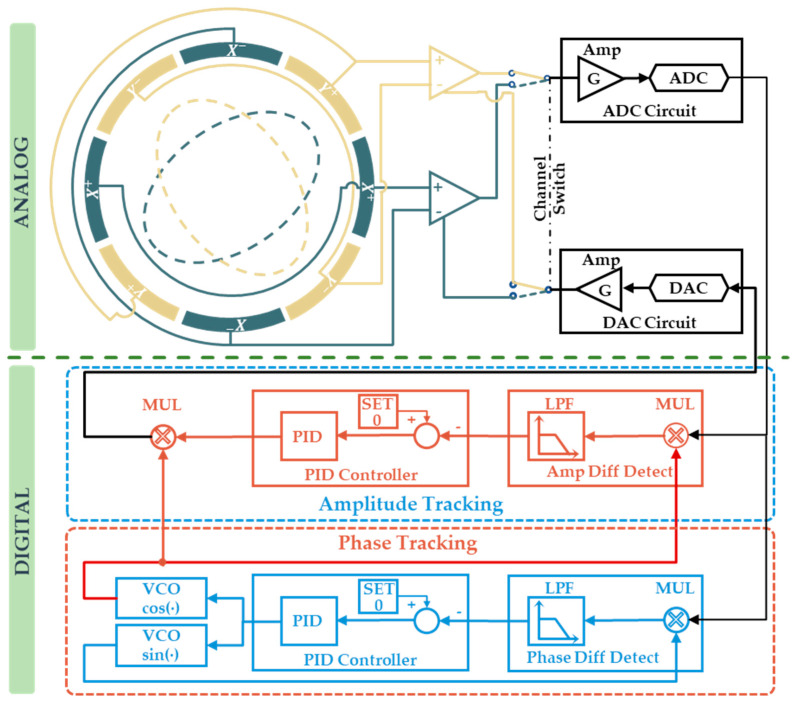
Closed-loop single-channel time division multiplexing circuit.

In this paper, a novel closed-loop single-channel time division multiplexing circuit is designed, as shown in Figure 2. The primary objective of closed-loop detection is to continuously monitor the vibration signal in real time within a closed-loop system, rather than sampling it directly in an open loop. In this way, all delays and gain drifts in the circuit can be eliminated by the closed-loop feature. Unlike conventional detection circuits, closed-loop detection circuits require digital controllers and DACs in addition to amplifiers. The ADC and DAC operate concurrently even during the vibration signal detection period. In terms of implementation, the DAC output signal is directly converged with the vibration signal during the detection period by switching the analog switch.

The most significant difference with the traditional method is that the vibration signal $A_{c}$ is not detected directly but is instead collected after the convergence of the signal $A_{e}$. This can be interpreted as acquiring error between the tracking signal output by the DAC and the actual vibration signal. The tracking signal is generated based on the error signal and solved by the digital controller. The loop forms a closed-loop control system with the control objective of suppressing the error signal to zero. Ultimately, the signal used for vibration state control is changed from $A_{c}$ to the tracking signal $D_{cf}$.

During the drive period, an analog switch interrupts the loop, allowing the DAC to exclusively output the control force calculated by the control loop without being influenced by the detection signal.

The actual vibration analog signal can be represented as follows:

(10) $$A_{c}\left(t\right)=a_{c}\cos\left(\omega_{\theta }t+\psi_{0}\right)$$

where $a_{c}$ is the amplitude of the vibration signal.

The following describes the signal transmission process and the tracking signal solving method. The tracking signal generated in the digital controller can be represented as follows:

(11) $$D_{fl}\left(t\right)=d_{fl}\cos\left(\omega_{\theta }t+\psi_{fl}\right)$$

where $d_{fl}$ is the amplitude of the tracking signal and $\psi_{fl}$ is the initial phase of the tracking signal.

$D_{fl}$ is changed to an analog signal after being output by the DAC and peripheral circuits. The analog tracking signal can be represented as follows:

(12) $$A_{f}\left(t\right)=K_{fb}d_{fl}\cos\left(\omega_{\theta }t+\psi_{fl}+\psi_{fb}\right)$$

where $K_{fb}$ and $\psi_{fb}$ are the gain and phase delay of the DAC and peripheral circuits.

The error signal resulting from the convergence of the two analog signals can be represented as follows:

(13) $$A_{e}\left(t\right)=a_{c}\cos\left(\omega_{\theta }t+\psi_{0}\right)-K_{fb}d_{fl}\cos\left(\omega_{\theta }t+\psi_{fl}+\psi_{fb}\right)$$

The error analog signal after amplification and ADC acquisition can be represented as follows:

(14) $$D_{e}\left(t\right)=K_{fw}a_{c}\cos\left(\omega_{\theta }t+\psi_{0}+\psi_{fw}\right)-K_{fw}K_{fb}d_{fl}\cos\left(\omega_{\theta }t+\psi_{fl}+\psi_{fb}+\psi_{fw}\right)$$

where $K_{fw}$ and $\psi_{fw}$ are the gain and phase delay of the ADC and peripheral circuits.

The generation of the tracking signal is mainly divided into two parts: tracking the phase of the vibration signal and tracking the amplitude of the vibration signal. The phase error and amplitude error can be obtained by I/Q demodulation of the error signal. Then the two errors are suppressed to zero by a closed loop to realize the tracking.

The phase difference between the error signal and the reference signal can be expressed as follows:

(15) $$\Delta \psi_{e}=LPF\left\{2D_{e}\left(t\right)\sin\left(\omega_{\theta }t+\psi_{fl}\right)\right\}=K_{fw}K_{fb}d_{fl}\sin\left(\psi_{fb}+\psi_{fw}\right)-K_{fw}a_{c}\sin\left(\psi_{0}+\psi_{fw}-\psi_{fl}\right)$$

The amplitude of the error signal can be represented as follows:

(16) $$\Delta D_{e}=LPF\left\{2D_{e}\left(t\right)\cos\left(\omega_{\theta }t+\psi_{fl}\right)\right\}=K_{fw}a_{c}\cos\left(\psi_{0}+\psi_{fw}-\psi_{fl}\right)-K_{fw}K_{fb}d_{fl}\cos\left(\psi_{fb}+\psi_{fw}\right)$$

By controlling $\Delta \psi_{e}$, $\Delta D_{e}$ to zero through closed-loop control, the relationship between the tracking signal and the actual vibration signal can be expressed as follows:

(17) $$\left\{\begin{array}{l} \psi_{fl}=\psi_{0}-\psi_{fb}\\ d_{fl}=\frac{a_{c}}{K_{fb}} \end{array}\right.$$

From the aforementioned equation, it is evident that the tracking signal remains unaffected by gain and phase delays during the signal acquisition process because the vibration signal and tracking signal are multiplexed within the acquisition circuit. Consequently, even if the acquisition circuit parameters vary, the gain and phase delay for both signals remain consistent, facilitating real-time tracking.

However, the tracking signal appears to be influenced by the DAC output circuit, as only the tracking signal passes through the DAC circuit without undergoing multiplexing. In fact, the multiplexing of the DAC circuit is not reflected in the detection period, but in the drive period. Taking the rate loop as an example, if a standing wave is driven to rotate at $\dot{\vartheta }$, according to Equations (4) and (8), the amplitude and modulation carrier of the actual calculated drive force can be expressed as follows:

(18) $$\left\{\begin{array}{l} f_{\vartheta }=-\frac{4\omega_{\theta }a\dot{\vartheta }}{K_{fb}}\\ R_{\vartheta }=2\cos(\omega_{\theta }t+\psi -\psi_{fb}) \end{array}\right.$$

When the drive period begins, the drive signal is converted into an analog signal through the DAC. After gain amplification and transmission delay, the actual drive signal applied to the resonator can be expressed as follows:

(19) $$\left\{\begin{array}{l} {\tilde{f}}_{\vartheta }=-4\omega_{\theta }a\dot{\vartheta }\\ {\tilde{R}}_{\vartheta }=2\cos(\omega_{\theta }t+\psi ) \end{array}\right.$$

Actual control aligns with expectations. The multiplexing of the DAC circuit during the detection and drive periods eliminates the influence of these circuits on vibration state control.

In summary, the closed-loop time division multiplexing circuit offers the following advantages. First, time division multiplexing allows *X*/*Y* signals to pass through the same analog circuit. The circuit gain and phase delay are completely consistent, eliminating the mismatch between different channels. Second, the impact of the phase delay is resolved by generating a real-time tracking signal. The final phase of the control signal applied to the resonator precisely matches the actual vibration phase. Third, the closed-loop detection circuit is not constrained to a unique operating point. When the external environment changes, such as temperature, the gain and phase delay of the circuit will drift. The tracking signal remains unaffected by the phase delay and gain drift.

## 4. Single-Channel Time Division Demodulation Error Analysis and Suppression

### 4.1. Single-Channel Signal Demodulation Error

**Figure 3:**
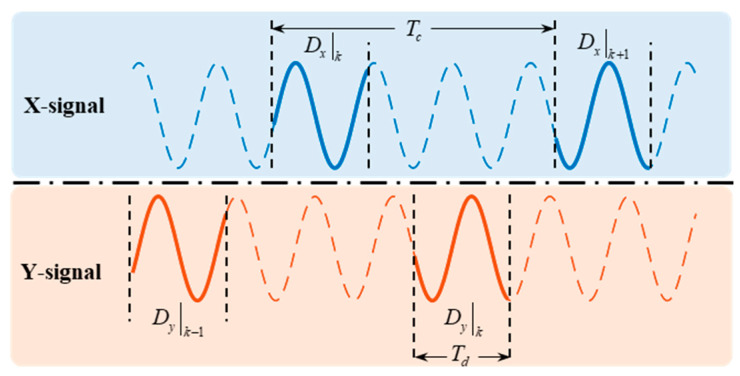
Time division *X*/*Y* channel detection signals.

Since the *X*/*Y* channel detection signals are separated in the time domain, the missing information leads to detection errors when the standing wave rotates. The schematic of the signal acquisition is illustrated in Figure 3.

A detection period $T_{c}$ can only acquire *X*/*Y* signals of time length $T_{d}$, which can be expressed as follows:

(20) $$\left\{\begin{array}{l} x\left(t_{x}\right)=a\cos\left[2\left(c\Omega t_{x}+\theta_{0}\right)\right]\cos\left(\omega t_{x}+\varphi_{0}\right)-q\sin\left[2\left(c\Omega t_{x}+\theta_{0}\right)\right]\sin\left(\omega t_{x}+\varphi_{0}\right)\\ y\left(t_{y}\right)=a\sin\left[2\left(c\Omega t_{y}+\theta_{0}\right)\right]\cos\left(\omega t_{y}+\varphi_{0}\right)+q\cos\left[2\left(c\Omega t_{y}+\theta_{0}\right)\right]\sin\left(\omega t_{y}+\varphi_{0}\right) \end{array}\right.$$

The acquisition periods $t_{x}$ and $t_{y}$ of the *X*/*Y* vibration signal can be expressed as follows:

(21) $$\left\{\begin{array}{l} t_{x}=\left[0\;\;T_{d}\right]+nT_{c}\\ t_{y}=\left[0\;\;T_{d}\right]+nT_{c}+\frac{T_{c}}{2} \end{array}\right.$$

where n∈ℕ represents the sampling sequence.

In this scheme, the *Y* channel signal consistently lags the *X* channel signal. In the implementation, the I/Q demodulation filter output of the final point of the $t_{x}$ period is utilized as the *X* channel demodulation result under the current sampling period, which is stored in a register. Similarly, the final point of the $t_{y}$ period is used as the *Y* channel demodulation result for this sampling. The *Y* channel result is combined with the *X* channel demodulation result stored in a register for the subsequent calculation. The reference signal for demodulation is generated independently of the sampling time within the FPGA, allowing it to be selected directly based on the actual *X*/*Y* channel sampling time. According to Equation (5), the primary demodulation result can be expressed as follows:

(22) $$\left\{\begin{array}{ll} C_{xn} & ={\left.LPF\left[V_{c}\left(t_{x}\right)x\left(t_{x}\right)\right]\right|}_{t_{x}=\left(T_{d}+nT_{c}\right)}=a\cos2\Theta \cos\left(\varphi -\varphi_{0}\right)+q\sin2\Theta \sin\left(\varphi -\varphi_{0}\right)\\ S_{xn} & ={\left.LPF\left[V_{s}\left(t_{x}\right)x\left(t_{x}\right)\right]\right|}_{t_{x}=\left(T_{d}+nT_{c}\right)}=a\cos2\Theta \sin\left(\varphi -\varphi_{0}\right)-q\sin2\Theta \cos\left(\varphi -\varphi_{0}\right)\\ C_{yn} & ={\left.LPF\left[V_{c}\left(t_{y}\right)y\left(t_{y}\right)\right]\right|}_{t_{y}=\left(T_{d}+nT_{c}+\frac{1}{2}T_{c}\right)}=a\sin\left(2\Theta +\Delta \Theta \right)\cos\left(\varphi -\varphi_{0}\right)-q\cos\left(2\Theta +\Delta \Theta \right)\sin\left(\varphi -\varphi_{0}\right)\\ S_{yn} & ={\left.LPF\left[V_{s}\left(t_{y}\right)y\left(t_{y}\right)\right]\right|}_{t_{y}=\left(T_{d}+nT_{c}+\frac{1}{2}T_{c}\right)}=a\sin\left(2\Theta +\Delta \Theta \right)\sin\left(\varphi -\varphi_{0}\right)+q\cos\left(2\Theta +\Delta \Theta \right)\cos\left(\varphi -\varphi_{0}\right)\\ \Theta & =c\Omega \left(T_{d}+nT_{c}\right)+\theta_{0}\\ \Delta \Theta & =c\Omega T_{c} \end{array}\right.$$

According to Equation (6), the secondary demodulation results can be expressed as follows:

(23) $$\left\{\begin{array}{ll} E_{n} & =a^{2}\cos^{2}2\Theta +q^{2}\sin^{2}2\Theta +a^{2}\sin^{2}\left(2\Theta +\Delta \Theta \right)+q^{2}\cos^{2}\left(2\Theta +\Delta \Theta \right)\\ Q_{n} & =2aq\cos\Delta \Theta \\ S_{n} & =\left(a^{2}-q^{2}\right)\sin4\Theta \cos\Delta \Theta +2\sin\Delta \Theta \left(a^{2}\cos^{2}2\Theta +q^{2}\sin^{2}2\Theta \right)\\ R_{n} & =a^{2}\cos^{2}2\Theta +q^{2}\sin^{2}2\Theta -a^{2}\sin^{2}\left(2\Theta +\Delta \Theta \right)-q^{2}\cos^{2}\left(2\Theta +\Delta \Theta \right)\\ L_{n} & =\sin2\left(\varphi -\varphi_{0}\right)\left[a^{2}\cos^{2}2\Theta +a^{2}\sin^{2}\left(2\Theta +\Delta \Theta \right)-q^{2}\sin^{2}2\Theta -q^{2}\cos^{2}\left(2\Theta +\Delta \Theta \right)\right] \end{array}\right.$$

Since the control frequency is typically less than 1 ms and the external angular velocity input is less than 10,000 degrees per second, ΔΘ is not greater than 0.05. Under the condition that the HRG has been closed-loop controlled, *q* is significantly smaller than *a.* The approximate relationship based on the above conditions can be expressed as follows:

(24) $$\left\{\begin{array}{l} \cos\Delta \Theta \approx 1\\ \sin\Delta \Theta \approx \Delta \Theta \\ \sin^{2}\Delta \Theta \approx 0\\ a^{2}\approx a^{2}+q^{2}\approx a^{2}-q^{2} \end{array}\right.$$

The demodulation results can be simplified as follows:

(25) $$\left\{\begin{array}{ll} E_{n} & \approx \left(a^{2}+q^{2}\right)\left(1+\Delta \Theta \sin4\Theta \right)=a^{2}\left(1+\Delta \Theta \sin4\Theta \right)\\ Q_{n} & \approx 2aq\\ S_{n} & \approx a^{2}\left(\sin4\Theta +\Delta \Theta \cos4\Theta +\Delta \Theta \right)\\ R_{n} & \approx a^{2}\left(\cos4\Theta -\Delta \Theta \sin4\Theta \right)\\ L_{n} & \approx a^{2}\sin2\left(\varphi -\varphi_{0}\right)\left(1+\Delta \Theta \sin4\Theta \right) \end{array}\right.$$

Time division error affects multiple loops within the system. As a critical output of HRG, the azimuth error can be expressed as follows:

(26) $$\theta_{e}=\frac{1}{4}\arctan\left(\frac{\sin4\Theta }{\cos4\Theta }\right)-\frac{1}{4}\arctan\left(\frac{S_{n}}{R_{n}}\right)\approx \frac{1}{4}\arctan\left(-\frac{\Delta \Theta \left(1+\cos4\Theta \right)}{1+\Delta \Theta \sin4\Theta }\right)$$

**Figure 4:**
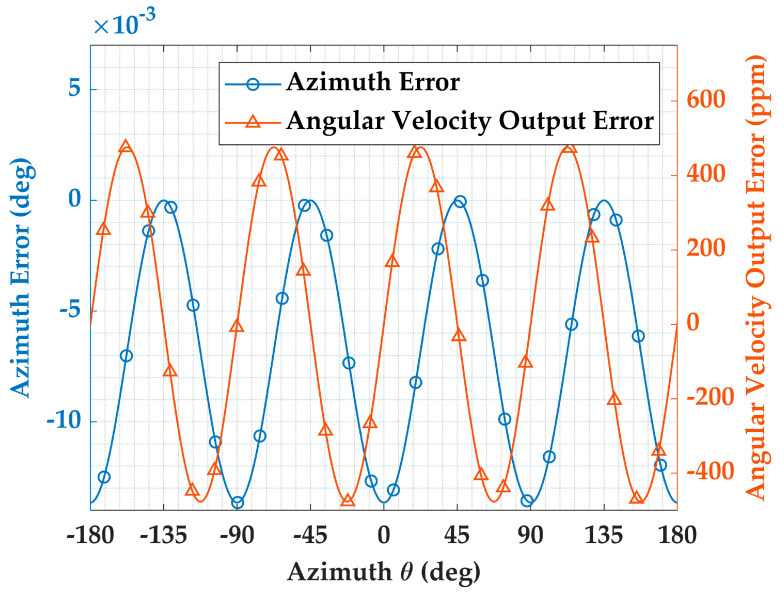
Azimuth solution error and angular velocity output error.

Taking a control period of 1 ms and an angular velocity input of 100 degrees per second as an example, a simulation is performed based on the equation above. The angle solution error and the angular velocity output error are shown in Figure 4. The angular solution error is minimized when the standing wave is near the *X*/*Y* axes because the vibration signal is predominantly distributed along one axis, with the other axis carrying almost no signal. As a result, even if there is a sampling time difference, the impact on the angle solution is relatively small. Similarly, the angular solution error reaches its maximum when the absolute values of the components of the two axes are nearly equal. The velocity output error is defined as the ratio of the absolute value of the velocity output error to the input angular velocity.

**Figure 5:**
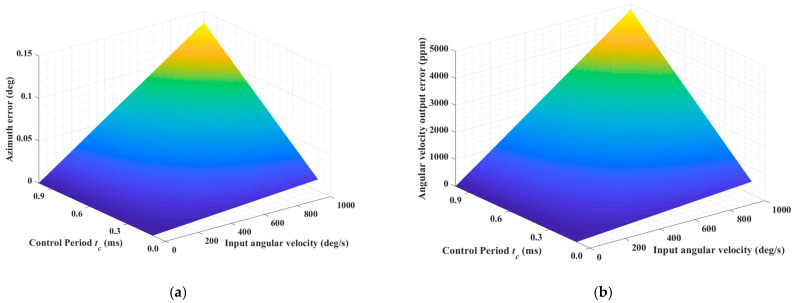
Maximum absolute value of the angular solution error and the angular velocity output error for different control periods and angular velocity inputs. (**a**) Maximum absolute value of the angular error; (**b**) maximum absolute value of the angular velocity output error.

Figure 5 gives the maximum absolute value of the angular solution error as well as the angular velocity output error for different control periods and angular velocity inputs.

### 4.2. Improved Single-Channel Time Division Demodulation

The errors in single-channel time division detection are determined by the angular velocity input and the current standing wave azimuth. Methods such as state observers can be employed to estimate the actual standing wave azimuth. However, these estimation methods are relatively complex and challenging to implement on embedded systems. An intuitive and computationally efficient error elimination method is described below. The detection error analyzed earlier is caused by the consistent delay of the *Y* channel relative to the *X* channel. The improved alternate demodulation method addresses this by alternating the delays between the *X*/*Y* channels through a doubling of the demodulation frequency. The acquisition time after raising the demodulation frequency can be expressed as follows:

(27) $$\left\{\begin{array}{l} {\tilde{t}}_{x}=\left[0\;\;T_{d}\right]+\frac{2m+1+{\left(-1\right)}^{m-1}}{4}T_{c}\\ {\tilde{t}}_{y}=\left[0\;\;T_{d}\right]+\frac{2m+1+{\left(-1\right)}^{m}}{4}T_{c} \end{array}\right.$$

where m∈ℕ represents the sampling sequence.

The primary demodulation result of alternate demodulation can be expressed as:

(28) $$\left\{\begin{array}{ll} C_{xm} & =a\cos2\tilde{\Theta }\cos\left(\varphi -\varphi_{0}\right)+q\sin2\tilde{\Theta }\sin\left(\varphi -\varphi_{0}\right)\\ S_{xm} & =a\cos2\tilde{\Theta }\sin\left(\varphi -\varphi_{0}\right)-q\sin2\tilde{\Theta }\cos\left(\varphi -\varphi_{0}\right)\\ C_{ym} & =a\sin\left(2\tilde{\Theta }+\Delta \tilde{\Theta }\right)\cos\left(\varphi -\varphi_{0}\right)-q\cos\left(2\tilde{\Theta }+\Delta \tilde{\Theta }\right)\sin\left(\varphi -\varphi_{0}\right)\\ S_{ym} & =a\sin\left(2\tilde{\Theta }+\Delta \tilde{\Theta }\right)\sin\left(\varphi -\varphi_{0}\right)+q\cos\left(2\tilde{\Theta }+\Delta \tilde{\Theta }\right)\cos\left(\varphi -\varphi_{0}\right)\\ \tilde{\Theta } & =c\Omega \left(T_{d}+\frac{2m+1+{\left(-1\right)}^{m-1}}{4}T_{c}\right)+\theta_{0}\\ \Delta \tilde{\Theta } & =\frac{{\left(-1\right)}^{m}}{2}c\Omega T_{c} \end{array}\right.$$

The most significant change is that the demodulation delay $\Delta \tilde{\Theta }$ alternates between positive and negative. According to Equation (25), the errors in the secondary demodulation results are all first-order terms of the demodulation delay $\Delta \tilde{\Theta }$. Therefore, the error is eliminated by averaging the results of two adjacent secondary demodulations to form a new demodulation sequence. Taking energy calculation as an example, the secondary demodulation result from alternate demodulation can be expressed as follows:

(29) $$\left\{\begin{array}{ll} E_{\tilde{n}} & =\frac{1}{2}\left(E_{m}+E_{m+1}\right)\\ & =\frac{E}{2}\left(1-\frac{1}{2}c\Omega T_{c}\sin\left[4c\Omega T_{d}+\left(l+1\right)4c\Omega T_{c}+\theta_{0}\right]+1+\frac{1}{2}c\Omega T_{c}\sin\left[4c\Omega T_{d}+\left(l+1\right)4c\Omega T_{c}+\theta_{0}\right]\right)\\ & =E\\ m & =2l+1 \end{array}\right.$$

where l∈ℕ represents the sampling sequence and $\tilde{n}$ is the new demodulation sequence.

## 5. Experiment

### 5.1. Phase Delay Elimination

**Figure 6:**
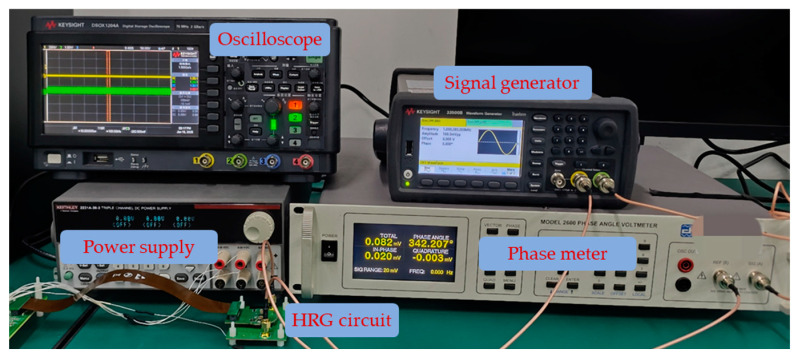
Experimental setup.

**Figure 7:**
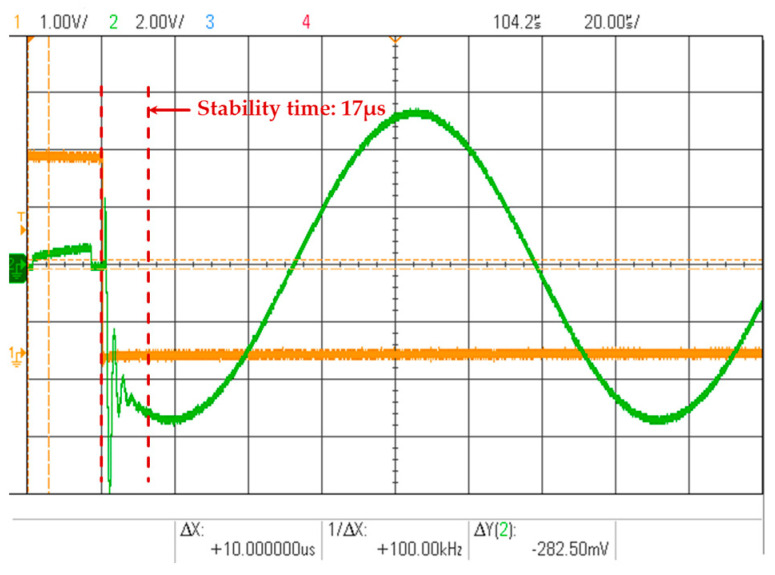
The establishment and stabilization process of tracking signals.

The experimental setup is shown in Figure 6. In the experiment, a signal generator is used to simulate the actual vibration signal, which is then fed as the input to the closed-loop single-channel circuit. As shown in Figure 7, the tracking signal for each detection period is observed through an oscilloscope. After a period of signal establishment, a stable sinusoidal tracking signal is output. In the actual calculation process, the signal establishment process needs to be discarded.

**Figure 8:**
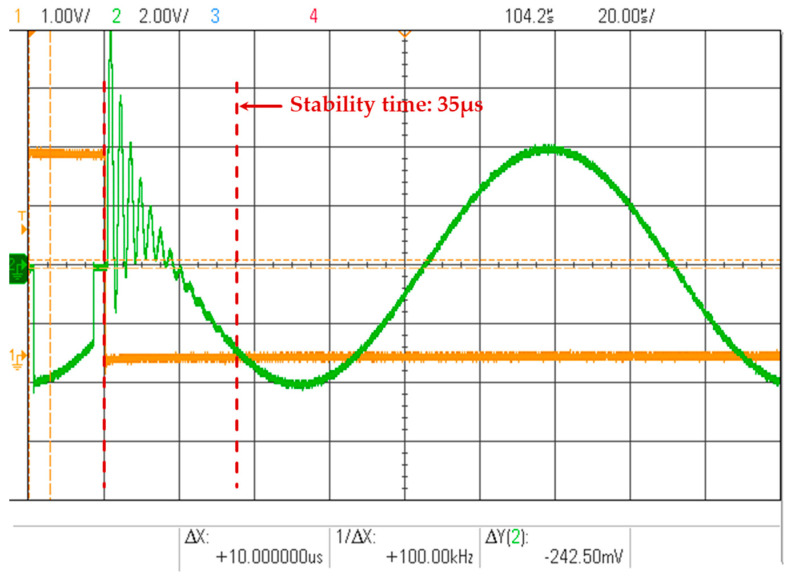
Tracking signals with additional loop phase delay 0.4 deg.

On the basis of the inherent phase delay in the loop, additional phase delay is introduced to test the phase difference between the tracking signal and the input signal. The results are summarized in Table 1. It is evident that even with the added phase delay, the tracking signal remains in phase with the input signal, unaffected by the inherent phase delay in the loop. However, the phase delay cannot be increased indefinitely. When the additional loop phase delay reaches 0.4 deg, the tracking signal establishment process, as shown in Figure 8, requires a duration of 35 μs to stabilize. In more extreme cases, when the system phase delay exceeds its phase margin, convergence cannot be achieved. Therefore, the tracking system must adapt its parameters based on the hardware constraints.

### 5.2. Gain Stability

Consider the integrator amplifier in the forward channel of a buffer circuit. By varying the amplifier gain, the amplitude tracking results are observed. As shown in Table 2, with different loop gains, the amplitude of the tracking signal remains stable and unchanged.

### 5.3. Angular Velocity Output Error

**Figure 9:**
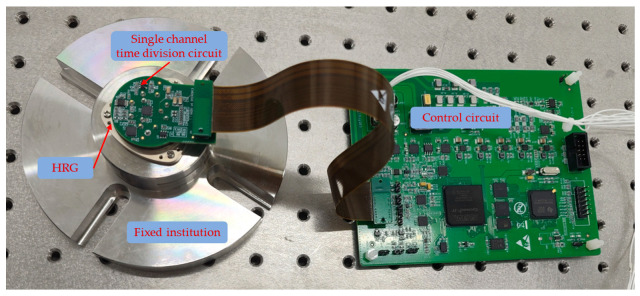
HRG and control circuit.

**Figure 10:**
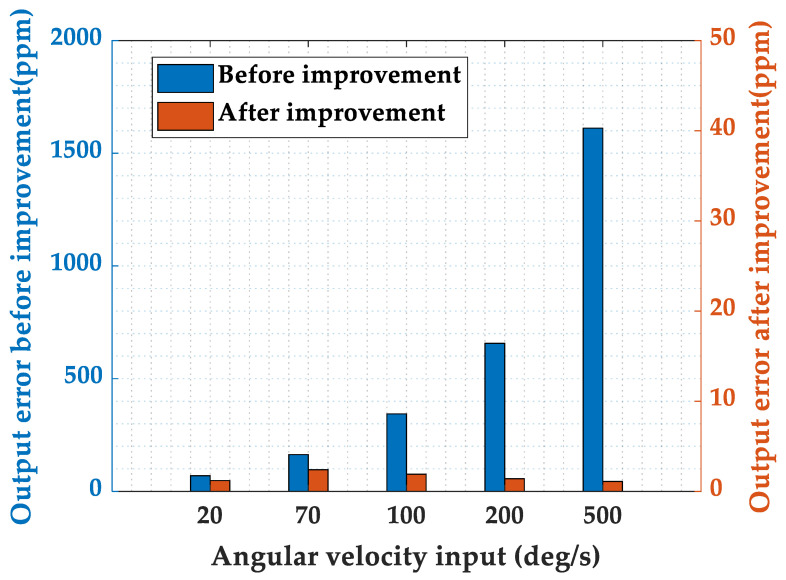
Comparison of the time division demodulation methods before and after improvement.

As shown in Figure 9, the HRG parameters used in the experiment are as follows: resonator diameter of 20 mm, resonance frequency of 7322 Hz, damping anisotropy of 2.3%, and frequency split of 1.7 mHz. A comparison of the time division demodulation methods before and after improvement is presented, with the output errors under different angular velocity inputs shown in Figure 10. As the angular velocity input increases, the output error before the improvement grows, consistent with the results observed in simulations. The residual errors after the improvement are primarily attributed to inherent manufacturing errors of the resonator, such as damping anisotropy and frequency split.

### 5.4. Allan Deviation

**Figure 11:**
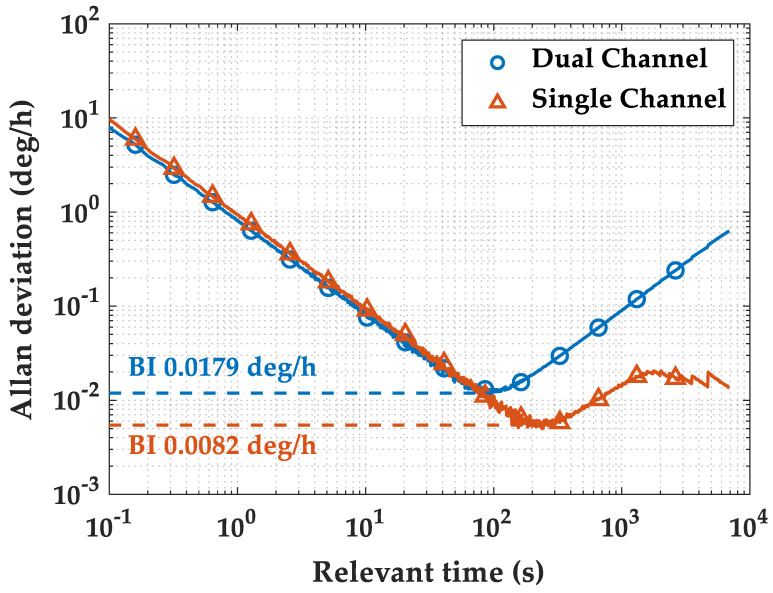
Allan deviation using single channel and dual channel.

As shown in Figure 11, the performance of the same HRG is tested using both the traditional dual-channel circuit and the closed-loop single-channel time division multiplexing circuit. The damping anisotropy has already been compensated. The fluctuations due to damping anisotropy are mainly the fourth harmonic of the standing wave azimuth. A sine–cosine function is chosen to Fourier fit the output fluctuations. The independent variable is the current standing wave azimuth, and the dependent variable is the damping drift of the current azimuth. The effect of damping anisotropy is eliminated in real time based on the current azimuth angle at the HRG output. The noise level in the single-channel circuit switch is slightly higher than that in the dual-channel system, but the tested gyroscope demonstrates better long-term stability.

## 6. Conclusions

A closed-loop single-channel detection circuit is designed to automatically suppress loop gain drift and phase delay. The time division multiplexing errors introduced by the system are optimized. This circuit eliminates the need for repeated calibration and compensation of circuit errors, as well as the necessity for complex real-time control algorithms for system identification. As a result, it ensures consistency and stability across different production batches, offering significant advantages in terms of system maintenance and scalability. This circuit can be widely applied to various types of Coriolis vibration gyroscopes and in different control modes.

## Figures and Tables

**Table 1:** Phase difference between tracking and input signals with additional phase delay.

Additional Phase Delay (Deg)	Phase Difference (Deg)
0.0	0.001
0.1	−0.001
0.2	0.002
0.3	0.001
0.4	−0.002
0.5	−0.001
0.6	−0.001

**Table 2:** Amplitude relative error with different loop gains.

Normalized Gain	Amplitude Relative Error (ppm)
1.0	5.2
1.2	−4.7
1.4	−4.9
1.6	3.3
1.8	3.7
2.0	3.3

## Data Availability

The original contributions presented in this study are included in the article. Further inquiries can be directed to the corresponding author.

## References

[1] Wei L., Kuai X., Bao Y., Wei J., Yang L., Song P., Zhang M., Yang F., Wang X. (2021). The Recent Progress of MEMS/NEMS Resonators. Micromachines.

[2] Zhang H., Zhang C., Chen J., Li A. (2022). A Review of Symmetric Silicon MEMS Gyroscope Mode-Matching Technologies. Micromachines.

[3] Perelyaev S.E. (2023). Current State of Wave Solid-State Gyroscopes. Development Prospects in Applied Gyroscopy. Proceedings of the 2023 30th Saint Petersburg International Conference on Integrated Navigation Systems (ICINS).

[4] Guérard J., Duquesnoy M., Le Traon O., Lavenus P., Ledier A.A., Levy R., Bonhomme J. (2024). The GYTRIX Quartz Crystal Axisymmetric Mems GYRO: Preliminary Measurements. Proceedings of the 2024 IEEE International Symposium on Inertial Sensors and Systems (INERTIAL).

[5] Lenoble A., Lenoir Y., Chenilleau J.-A., Morin P., Baudry H. (2023). Navkite^TM^—Protection of Inertial Navigation System Based on Interference Detection and Mitigation Solution. Proceedings of the 2023 DGON Inertial Sensors and Systems (ISS).

[6] Wei J., Yu F., Zhang Y., Fan S., Chang L., Guo K. A System-Level Calibration and Integrated Navigation Technology of HRG-Based SINSDVL System for Underwater Vehicles. Proceedings of the 2023 IEEE International Conference on Mechatronics and Automation (ICMA).

[7] Seok J., Kim Y.-J., Jang K.-I., Min B.-K., Lee S.J. (2007). A Study on the Fabrication of Curved Surfaces Using Magnetorheological Fluid Finishing. Int. J. Mach. Tools Manuf..

[8] Basarab M.A., Lunin B.S., Matveev V.A., Chumankin E.A. (2015). Balancing of Hemispherical Resonator Gyros by Chemical Etching. Gyroscopy Navig..

[9] Choi S.-Y., Kim J.-H. (2011). Natural Frequency Split Estimation for Inextensional Vibration of Imperfect Hemispherical Shell. J. Sound Vib..

[10] Wei Z., Yi G., Huo Y., Xi B., Zhao Y. (2021). High-Precision Synchronous Test Method of Vibration Performance Parameters for Fused Quartz Hemispherical Resonator. Measurement.

[11] Huo Y., Wei Z., Ren S., Yi G. (2023). High Precision Mass Balancing Method for the Fourth Harmonic of Mass Defect of Fused Quartz Hemispherical Resonator Based on Ion Beam Etching Process. IEEE Trans. Ind. Electron..

[12] Yuan L., Zeng Q., Wang C., Ren S., Wei Z., Huo Y. (2023). High Precision Assembly and Welding Method for Flat-Electrode Hemispherical Resonator Gyro. IEEE Trans. Instrum. Meas..

[13] Fan Q., Zhou Y., Liu M., Lin C., Su Y. (2021). Rate-Integration Gyroscope (RIG) With Gain Self Calibration. IEEE Sens. J..

[14] Vatanparvar D., Shkel A.M. (2021). Identification of Gain Mismatches in Control Electronics of Rate Integrating CVGs. Proceedings of the 2021 IEEE International Symposium on Inertial Sensors and Systems (INERTIAL).

[15] Chen W., Ding X., Qin Z., Ge X., Li H. (2024). Identification and Compensation of Gain Mismatches for Whole-Angle Microhemispherical Resonator Gyroscope Based on Modal Reversal. IEEE Sens. J..

[16] Sun Y., Wei Z., Yi G., Wang N. (2024). Identification and Compensation Method of Unbalanced Error in Driving Chain for Rate-Integrating Hemispherical Resonator Gyro. Sensors.

[17] Sun J., Yu S., Zhang Y., Shi Y., Lu K., Xi X., Wu X., Xiao D. (2022). Characterization and Compensation of Detection Electrode Errors for Whole-Angle Micro-Shell Resonator Gyroscope. J. Microelectromech. Syst..

[18] Sun J., Liu K., Yu S., Zhang Y., Xi X., Lu K., Shi Y., Wu X., Xiao D. (2022). Identification and Correction of Phase Error for Whole-Angle Micro-Shell Resonator Gyroscope. IEEE Sensors J..

[19] Tang X., Pan Y., Zeng L., Li J., Tao Y., Jia Y., Yang K., Luo H. (2022). Investigation on Influences of Phase Delay on Performance of Resonator Gyroscopes. Proceedings of the 2022 DGON Inertial Sensors and Systems (ISS).

[20] Wang J., Yang G., Zhou Y., Zhang J., Liu F., Cai Q. (2024). An In-Run Automatic Demodulation Phase Error Compensation Method for MEMS Gyroscope in Full Temperature Range. Micromachines.

[21] Tang X., Zeng L., Deng K., Li J., Xiao P., Xia T., Pan Y., Luo H. (2024). A Rate Hemispherical Resonator Gyroscope with Bias Stability of 0.0056°/h with Compensation of Phase Delay. Sens. Actuators A Phys..

[22] Xu R., Gao Z., Nan F., Zhang Y. (2021). Single-Channel Control for Hemispherical Resonator Gyro Based on Time Division Multiplexing and Demultiplexing. IEEE Sensors J..

[23] Xu R., Gao Z., Nan F., Xu X., Du D., Zhang Y. (2024). Control System Based on Double-Frequency Drive and Single-Channel Detection for Rate Integrating Vibratory Gyroscope. IEEE Sens. J..

[24] Sun Y., Wei Z., Yi G., Xi B. (2023). A Rate Integrating Hemispherical Resonant Gyro Angular Rate Output Method Based on Frequency Demodulation. Proceedings of the 2023 2nd International Symposium on Sensor Technology and Control (ISSTC).

[25] Ding X., Ruan Z., Pu Y., Zhang H., Li H., Zhao L. (2022). Bias Modulation of Force-to-Rebalanced Micro Hemispherical Resonator Gyroscope Based on Mode-Rotation. IEEE Sensors J..

[26] Zhao W., Yang H., Liu F., Su Y., Li C. (2021). High Sensitivity Rate-Integrating Hemispherical Resonator Gyroscope with Dead Area Compensation for Damping Asymmetry. Sci. Rep..

[27] Zhang X., Li P., Zhuang X., Sheng Y., Liu J., Gao Z., Yu Z. (2023). Weak Capacitance Detection Circuit of Micro-Hemispherical Gyroscope Based on Common-Mode Feedback Fusion Modulation and Demodulation. Micromachines.

[28] Xie J., Zhou T., Li X., Chen Y., Yang T., Su Y. (2025). A MEMS-Based Three-Ring Capacitive Angular Position Sensor with an Absolute Zero Position Feature. IEEE Trans. Ind. Electron..

